# HIV-1 Treated Patients with Undetectable Viral Loads have Lower Levels of Innate Immune Responses via Cytosolic DNA Sensing Systems Compared with Healthy Uninfected Controls

**DOI:** 10.4172/2155-6113.1000315

**Published:** 2014-06-10

**Authors:** Sanjay Swaminathan, Hongyan Sui, Joseph W Adelsberger, Qian Chen, Michael Sneller, Stephen A Migueles, Shyamasundaran Kottilil, Alexander Ober, Sara Jones, Catherine A Rehm, H Clifford Lane, Tomozumi Imamichi

**Affiliations:** 1 Applied and Developmental Research Directorate, Leidos Biomedical Research Inc., Frederick National Laboratory for Cancer Research (FNLCR), Frederick, MD 21702, USA; 2 Department of Clinical Immunology, Western Sydney Local Health District, Sydney, Australia; 3 Sydney Medical School, University of Sydney, Sydney, Australia; 4 School of Medicine, University of Western Sydney, Sydney, Australia; 5 Laboratory of Immunoregulation, National Institute of Allergy and Infectious Diseases, National Institutes of Health, Bethesda, MD 20892, USA; 6 Clinical Research Program Directorate, Leidos Biomedical Research, Inc., FNLCR, Frederick, MD 21702, USA

**Keywords:** HIV-1, DNA sensing, Innate immunity, Cytosolic DNA sensors, DNA virus, IFN-λ1, IFN-β, RANTES

## Abstract

**Objectives:**

After DNA or RNA virus infection, cytosolic foreign DNA or RNA derived from the infecting viruses is recognized by intracellular pathogen recognition receptors (PRRs) and induces activation of the innate immune system. Transfection of DNA has been used as an experimental model for DNA virus-mediated innate responses. We have previously reported that DNA transfection preferentially induces Type-III IFN (IFN-λ1) rather than Type-I IFN (IFN-β). In this study, we compared the DNA-mediated immune response between healthy controls and HIV-1 infected patients with undetectable viral loads and assessed potential innate immune responses in these patients.

**Methods:**

The study consisted of 50 HIV-1 negative healthy donors, 46 patients on combination antiretroviral therapy with HIV-1 viral loads <50 copies/ml and 7 long term non-progressors (LTNPs). PBMCs were isolated from whole blood using Ficoll-Paque. DNA transfection was performed using Lipofectamine 2000. After 22 hours incubation, total cellular RNA was extracted and real time RT-PCR was performed to determine gene expression level of IFN-λ1, IFN-β and RANTES. Gene induction was compared by fold change.

**Results:**

Baseline levels of endogenous gene expression of IFN-λ1, IFN-β and RANTES in HIV-1 patients were higher than in controls. Following DNA transfection, both HIV infected patients and healthy controls induced gene induction, however, the induction in HIV-1 patients was at a significantly lower level compared to uninfected controls.

**Conclusion:**

HIV-1 treated patients with undetectable viral loads have lower levels of innate immune responses via cytosolic DNA sensing systems. This may be caused by persistent immune activation.

## Introduction

Triggering of innate immune responses relies on the detection of pathogen associated molecular patterns(viral DNA, RNA, endotoxin, flagellin, lipoteichoic acid and peptidoglycan) by pattern recognition receptors (PRRs) in host cells [1]. A number of PRRs for foreign DNA have been identified and typically belong to members of the Toll-like receptor (TLR) family of membrane bound DNA sensors [2] near 10. cytosolic DNA sensors [3-9]. The result of foreign DNA being detected by these PRRs leads to induction of IFN-β or activation of Caspase I [10]. Our previous work has demonstrated for the first time that IFN-λ1 (also known as IL-29) is also highly induced following a DNA virus infection or DNA transfection into primary human macrophages, dendritic cells and some cell lines [11]. This induction was mediated by Ku70, a protein related with DNA repair.

It is well recognized that HIV-1 infected patients who are virally suppressed on treatment, have persistent and significantly higher level of immune activation compared to uninfected controls [12]. It is not known whether this persistent immune activation is associated with changes in innate immunity in response to invading foreign DNA. The aim of this study was to investigate whether there were differential immune responses in the form of IFN gene expression following DNA transfection between virally suppressed patients treated with cART and uninfected controls.

## Methods

### 

#### Isolation of cells

Under ethics approval by the institutional review board of the National Institute of Allergy and Infectious Diseases, a total of 50 healthy controls, 46 HIV-1 infected patients on cART with HIV-1 viral loads<50 copies/ml and 7 LTNPs were used in this study. Patients with cART were administrated with nucleoside reverse transcriptase (RT) inhibitors (lamivudine, abacavir or emtricitabine), nucleotide RT inhibitor (tenofovir), non-nucleoside RT inhibitors (nevirapine or efavirenz), protease inhibitors (ritonavir, atazanavir or darunavir) or integrase inhibitor (raltegravir). None of the patients, however, were on CCR5 antagonist, maraviroc (supplementary Table 1). The mean CD4+ T cell counts for the virally suppressed patients and LTNP were 569 cells/microlitre (range 98-1165 cells/μl, median: 584 cells/μl) and 569 cells/ml (range 307-963 cells/μl, median: 480 cells/μl), respectively (supplementary Table 1). The mean period of time before enrollment in which plasma viral loads have been consistently under 50 RNA copies/ml was 388 weeks (range 16-741 weeks). PBMCs were isolated from 20 mL of heparinized whole blood using Ficoll-Paque [13]. Purified populations of CD3+/CD4+ and CD3-/CD14+ cells were obtained by means of FACS-sorting with the BD FACSAria II SORP (BD Biosciences) cell sorter. CD4+ T cells and monocytes were enriched prior to FACS sorting by using Human CD4+ T cell and Human Monocyte Enrichment Kit (Stemcell Technologies), respectively. Sort purity was usually >99% for each CD3+/CD4+ and CD3-/CD14+ cells subset.

### DNA transfection and gene detection

A non-coding plasmid DNA (pCR2.1, Life Technologies) was isolated using Endofree plasmid kit (Qiagen) and digested with EcoR1 (Roche) to make linearized DNA. The digested DNA was purified using PCR isolation kit (Qiagen). DNA transfection was performed using Lipofectamine 2000 (Life Technologies). Cells (3 million at 1×10^6^/ml) were transfected with 3μg of linearized DNA for 22 hours in RPMI-1640 (Invitrogen) with 10 % FBS (Hyclone) in a 6 well plate. After incubation, cellular RNA was extracted using the RNeasy Mini kit (Qiagen) and real time qRT-PCR was performed using Taqman probes for IFN-λ1, IFN-β, RANTES and GAPDH (Applied Biosystems) with C1000 Thermal Cycler (BioRad). Fold change of gene products was calculated using the delta-delta CT method [11].

### Statistical analysis

Differences in cytokine gene expression between healthy control and HIV infected patients were determined by using unpaired *t* test (Prism 6 for Windows) (Graph Pad).

## Results

**Figure 1:**
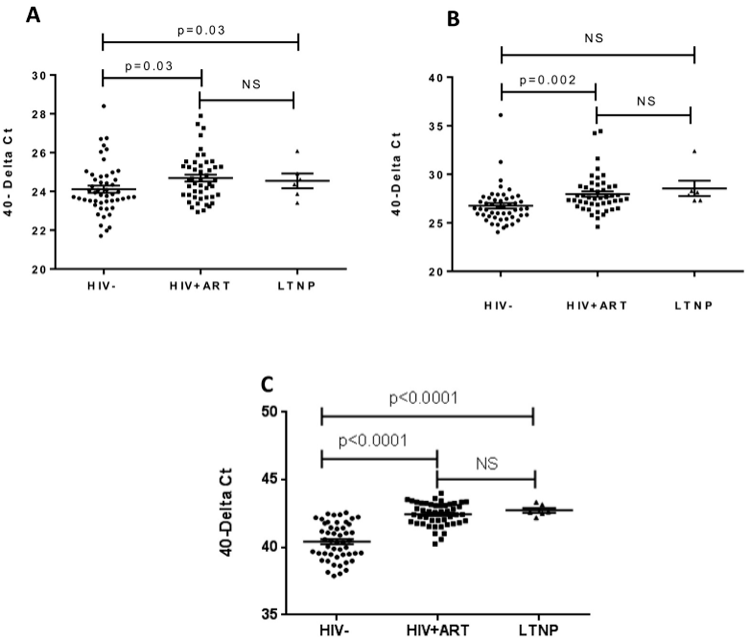
Comparison of baseline gene expression in PBMCs PBMCs from three patient groups (HIV negative controls, HIV+ ART patients, and LTNPs) were treated with 6 μL of Lipofectamine 2000 (mock transfection) for 22 hours. Total RNA was extracted and subjected to qRT-PCR. Baseline gene expression levels of IFN-λ1 (A), IFN-β (B) and RANTES (C) were calculated by comparing the Ct values of the gene of interest with the Ct value of GAPDH and subtracting this from 40 (40-delta Ct).

Freshly un-stimulated PBMCs were treated with mock (lipid alone) or DNA transfection, and then gene expression was monitored using real time RT-PCR. To compare baseline gene expression levels of IFN-λ1, IFN-β and RANTES, Ct value of each of gene was calculated by comparing with the Ct value of GAPDH in mock-transfected PBMCs. To avoid misreading the data, the number was subtracted from 40 (normalized gene expression: 40-dCt). This revealed a small but significant (p=0.03) elevation in baseline IFN-λ1 gene expression in the HIV+ cART group (24.7 ± 0.17) compared to controls (24.1 ± 0.18), however the gene expression was not significantly changed between HIV+cART and LNTP (Figure 1A), indicating that these genes are endogenously activated in HIV+cART and LTNP. Baseline gene expression of IFN-β (Figure 1B) and RANTES (Figure 1C) were also significantly higher in HIV-1+cART patients compared to controls, suggesting persistent immune activation was responsible for the gene activation observed in basal level. Of interest, It looks like basal level of IFN-lambda 1 and RANTES gene activation in LTNPs was relatively higher than healthy controls from Figure 1.

**Figure 2:**
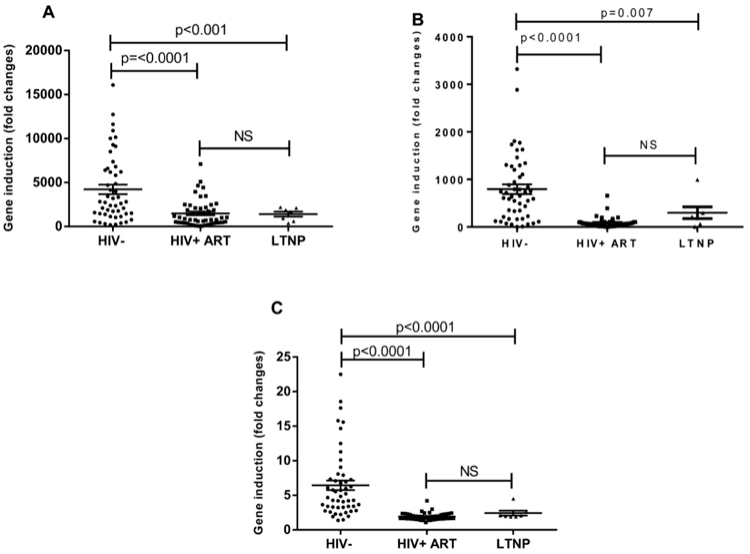
Measurement of IFN-λ1, IFN-β and RANTES gene induction following DNA transfection into PBMCs PBMCs from three patient groups (HIV negative controls, HIV+ ART patients and LTNP) were either mock transfected (lipid alone) or transfected with 3 ug of linearized plasmid non-coding plasmid DNA using Lipofectamine 2000 and left for 22 hours. Total RNA was extracted and subjected to qRT-PCR. The amount of gene induction following DNA transfection was calculated for IFN-λ1 (A), IFN-β (B) and RANTES (C) by comparing gene expression levels from DNA transfected PBMCs with that of mock transfected PBMCs (using the delta-delta Ct method).

Transfection of DNA into PBMCs in control (healthy donors) subjects showed 4223.0 ± 540 (Mean + SE) fold increase in FN-lambda1 gene expression compared to the basal level of the gene expression (Figure 2A). In patients with treated HIV-1 infection and LTNP, the mean fold gene expression increase was 1491 ± 215 and 1404 ± 282, respectively and this was significantly lower compared to uninfected controls (p<0.0001). The fold increases in IFN-β gene expression following DNA transfection into PBMCs in uninfected controls was 793.4 ± 100 (Figure 2B), while in HIV-1 cART treated patients and LTNPs, they were 84.9 ± 15 and 298 ± 124 respectively. Following DNA transfection in PBMCs from uninfected controls, there was a 6.45 ± 0.69 fold increase in RANTES gene expression (Figure 2C), while in HIV-1 treated patients and LTNP, the gene expression were induced by 1.9 ± 0.072 and 2.4 ± 0.36 fold, respectively (p<0.0001).

## Discussion

It is known that invading foreign cytoslic DNA by virus infection or DNA transfection induces Type-I or Type-III Interferons as an innate immune response via host DNA sensors [5-7]. We compared the DNA-mediated innate immune response between healthy controls and HIV infected patients on cART with HIV load <50 copies/ml using whole PBMC One of our goals in this study was to define whether or not PBMC responds to the invading DNA and then induce innate response genes. Although the HIV viral load was below the detection level, the induction level of genes in the patients was significantly lower in HIV-1 infected patients compared to healthy controls. Of note, those genes that were endogenously activated in HIV-1 infected patients, sub-optimally responded to DNA transfection which we believe is due to the persistent immune activation that results in reduced innate immune responses following DNA challenge.

Since we observed a lower level of immune responses in ART-treated patients, it was assumed that the ART might be a factor that leads to persistent immune activation. However, LTNPs(antiretroviral-naïve, who has not been treated with ART) also demonstrated the lower level of innate immune response following DNA transfection, implying that integrated proviral DNA or uncharacterized cellular factors rather than antiviral drugs may be involved in the persistent immune activation in chronically infected patients. Recent studies reported the cytosolic accumulation of incomplete HIV reverse transcripts (proviral DNA) in acute HIV-infection induces innate immune responses [14,15]. These viral DNAs are sensed by IFN-γ-inducible protein 16 (IFI16) or cyclic guanosine monophosphate-adenosine monophosphate (cGAMP) leading to caspase-1 activation and pyroptosis in T cells [14] or IFN-responses in cell line [15]. The reports illustrated drug treatment naïve patients may also have a persistent immune activation in acute infection, however, a more recent study reported that innate DNA sensing by cGAS and IFI16 in T cells is impaired in chronically infected HIV patients [16,17], suggesting that mechanisms behind the innate immune activation in acute infection may differ from that in chronic infection.

IFN-λ1 and IFN-β are induced by monocytes, macrophages and dendritic cells [18], and those cells express cytosolic DNA sensor proteins. Around 10 cytosolic DNA sensor proteins associated with immune activation have been identified [3-9]. We have previously reported that DNA-virus infection or DNA transfection (a model of DNA-virus infection) induces IFN-λ1. in primary macrophages, dendritic cells and some cell lines [11,19], and Ku70, a DNA repair protein, serves as a DNA sensor protein for the induction [11]. In the current study, we demonstrated that the DNA-mediated IFN-λ1 induction in HIV infected patients is significantly lower than healthy uninfected controls. To define which cell types in PBMCs are involved in the DNA-mediated gene induction, we transfected DNA into sorted cells and gene activation was analyzed. Monocytes (CD3-/CD14+) but not T cells induced IFN-λ1, IFN-β and RANTES genes (Supplementary Figure 1), As shown in Supplementary Table 1, which shows total CD4+ T cell counts in HIV-1 infected patients on ART, only one patient had a CD4+ T cell count below 100 and only one other patient had a CD4+ T cell count <200. The mean CD4+ T cell count was 592 cells/μl and the median count was 584 cells/μl, reflecting that the majority of patients had relatively well preserved CD4+ T cell counts. A statistical analysis demonstrated that there is no significant correlation between CD4+ T cell counts and activation of each gene (Supplementary Figure 2). Therefore it appeared likely that the lower level of innate response was derived from monocytes/macrophages rather than T-cells. One may predict that the number of monocytes in HIV infected patients was lower than uninfected controls, which may explain the lower innate immune responses observed. However, Krishnan et al., have reported that LTNPs have significantly higher proportion of CD14+ monocytes compared to HIV negative control [20], thus the lower level of immune response in drug naïve is probably not cell number dependent. To the best of our knowledge, this is the first report demonstrating that DNA-mediated Type-III IFN induction is lower in either HIV infected patients with ART or LTNPs. Further work is required to precisely understand how the persistent immune activation is induced and how this may affect innate immune responses.

## Conclusion

We have demonstrated for the first time that HIV infected patients with viral loads <50 copies/ml are lower responders to foreign DNA as measured by innate immune gene responses. Our findings may provide new insight into the immune dysfunction observed in HIV-1 infected patients, despite optimal antiviral treatment leading to viral load suppression.

## Supplementary Material

Supplementary file
