# Supplementary material for: HIV-1 Treated Patients with Undetectable Viral Loads have Lower Levels of Innate Immune Responses via Cytosolic DNA Sensing Systems Compared with Healthy Uninfected Controls
Source: J AIDS Clin Res. Author manuscript; Available in PMC 2015 May 26. (PMC4444065; doi:10.4172/2155-6113.1000315)
Supplement: Supplementary file [file NIHMS624682-supplement-Supplementary_file.zip › Supplemental table1.v4.docx]

**Supplemental table 1**: Demographics of HIV-1 infected patients and drugs used for viral suppression

| **Pt^1^** | **Sex** | **Drugs Used^2^** | **Age** | **CD4%^3^** | **T.CD4^4^** | **Weeks^5^** |
| --- | --- | --- | --- | --- | --- | --- |
| 1 | M | AZV/RTV/FTC/TDV | 39 | 27 | 512 | 208 |
| 2 | M | AZV/RTV/FTC/TDV | 32 | 30 | 219 | 26 |
| 3 | M | 3TC/RTV/DRV/RAL | 61 | 21 | 289 | 569 |
| 4 | M | AZV/RTV/TDV/ABC/3TC | 54 | 24 | 393 | 429 |
| 5 | M | ABC/EFV/FTC/TDV | 49 | 35 | 851 | 416 |
| 6 | M | EFV/FTC/TDV | 50 | 37 | 256 | 741 |
| 7 | M | NVP/TDV | 54 | 34 | 638 | 308 |
| 8 | M | EFV/FTC/TDV | 39 | 14 | 98 | 30 |
| 9 | M | NVP/FTC/TDV | 47 | 40 | 579 | 459 |
| 10 | F | EFV/FTC/TDV | 51 | 12 | 160 | 16 |
| 11 | M | EFV/FTC/TDV | 77 | 48 | 615 | 359 |
| 12 | M | RIT/FTC/TDV/DRV | 59 | 22 | 470 | 156 |
| 13 | M | RIT/ABC/3TC/DRV | 49 | 24 | 369 | 490 |
| 14 | M | AZV/ABC/3TC | 55 | 35 | 662 | 698 |
| 15 | M | AZV/RTV/FTC/TDV | 61 | 59 | 773 | 468 |
| 16 | F | AZV/RTV/FTC/TDV | 46 | 32 | 740 | 598 |
| 17 | F | AZV/RTV/FTC/TDV | 57 | 37 | 865 | 139 |
| 18 | F | RIT/ABC/3TC/DRV | 47 | 25 | 592 | 459 |
| 19 | M | RIT/FTC/TDV | 59 | 39 | 688 | 706 |
| 20 | M | AZV/RTV/FTC/TDV/ABC/3TC | 52 | 24 | 357 | 160 |
| 21 | M | EFV/TDV/ABC/3TC | 50 | 44 | 1092 | 533 |
| 22 | F | EFV/FTC/TDV | 53 | 30 | 666 | 156 |
| 23 | M | ABC/3TC/DRV | 47 | 30 | 303 | 43 |
| 24 | M | RTV/FTC/TDV/DRV | 33 | 28 | 403 | 91 |
| 25 | M | EFV/FTC/TDV | 49 | 37 | 1024 | 702 |
| 26 | M | AZV/RTV/FTC/TDV | 53 | 38 | 551 | 195 |
| 27 | M | AZV/RTV/FTC/TDV | 42 | 27 | 348 | 217 |
| 28 | M | EFV/FTC/TDV | 48 | 46 | 856 | 386 |
| 29 | M | EFV/FTC/TDV | 32 | 46 | 1165 | 286 |
| 30 | M | AZV/RTV/ABC/3TC | 39 | 32 | 800 | 195 |
| 31 | M | RTV/AZV/FTC/TDV | 44 | 23 | 570 | 585 |
| 32 | M | EFV/FTC/TDV | 33 | 24 | 543 | 126 |
| 33 | M | EFV/FTC/TDV | 63 | 34 | 589 | 152 |
| 34 | M | EFV/FTC/TDV | 54 | 42 | 757 | 303 |
| 35 | M | EFV/FTC/TDV | 57 | 49 | 686 | 641 |
| 36 | M | EFV/FTC/TDV | 43 | 30 | 479 | 286 |
| 37 | M | AZV/RTV/TDV/NVP | 56 | 38 | 590 | 286 |
| 38 | M | AZV/RTV/ABC/3TC | 52 | 18 | 358 | 598 |
| 39 | M | AZV/RTV/FTC/TDV | 41 | 36 | 565 | 130 |
| 40 | M | EFV/FTC/TDV | 43 | 44 | 863 | 468 |
| 41 | M | AZV/RTV/FTC/TDV | 50 | 29 | 377 | 706 |
| 42 | M | RTV/FTC/TDV/DRV/RAL | 65 | 25 | 613 | 685 |
| 43 | M | EFV/FTC/TDV | 58 | 40 | 1159 | 633 |
| 44 | M | EFV/FTC/TDV | 53 | 30 | 540 | 342 |
| 45 | F | EFV/FTC/TDV | 32 | 31 | 772 | 672 |
| 46 | M | EFV/FTC/TDV | 65 | 29 | 424 | 641 |
| L1 | F | N/A | 34 | 26 | 307 | N/A |
| L2 | F | N/A | 49 | 17 | 403 | N/A |
| L3 | M | N/A | 52 | 32 | 557 | N/A |
| L4 | M | N/A | 42 | 32 | 370 | N/A |
| L5 | F | N/A | 63 | 52 | 963 | N/A |
| L6 | M | N/A | 52 | 45 | 814 | N/A |
| L7 | M | N/A | 45 | 45 | 981 | N/A |

1: 1-46: HIV treated patients, L1-L7: Long Term Non-progressors. 2: 3TC = lamuvidine; ABC = abacavir; FTC = emtricitabine; NVP = nevirapine; EFV = efavirenz; TDV = tenofovir; AZV = atazanavir; DRV = darunavir; RTV = ritonavir; RAL = raltegravir, 3: Percentage of total CD4 in whole blood cells, 4: total CD4+ T cell counts (cells/μl), 5: Weeks of viral load <50 on cART.
