# Supplementary material for: HIV-1 Treated Patients with Undetectable Viral Loads have Lower Levels of Innate Immune Responses via Cytosolic DNA Sensing Systems Compared with Healthy Uninfected Controls
Source: J AIDS Clin Res. Author manuscript; Available in PMC 2015 May 26. (PMC4444065; doi:10.4172/2155-6113.1000315)
Supplement: Supplementary file [file NIHMS624682-supplement-Supplementary_file.zip › supplementary figure legned.docx]

**Supplemental Figure 1**. **Measurement of IFN-λ1, IFN-β and RANTES gene induction following DNA transfection into sorted cells.** Sorted cells from normal PBMCs were either mock transfected (lipid alone) or transfected with 1 µg of linearized plasmid non-coding plasmid DNA using Lipofectamine 2000 and left for 22 hours. Total RNA was extracted and subjected to qRT-PCR. The amount of gene induction following DNA transfection was calculated for IFN-λ1, IFN-β and RANTES by the delta-delta Ct method. Data shows means+SD from a representative result from three independent experiments.

**Supplemental Figure 2.Correlation of CD4 T cell counts and gene induction**

Levels of CD4+ T cell counts and gene induction of IFN-λ1 (A), IFN-β (B) or RANTES (C) in each patient (HIV+cART) were plotted. No correlation was observed.
