# Supplementary figures and images for: HIV-1 Treated Patients with Undetectable Viral Loads have Lower Levels of Innate Immune Responses via Cytosolic DNA Sensing Systems Compared with Healthy Uninfected Controls
Source: J AIDS Clin Res. Author manuscript; Available in PMC 2015 May 26. (PMC4444065; doi:10.4172/2155-6113.1000315)

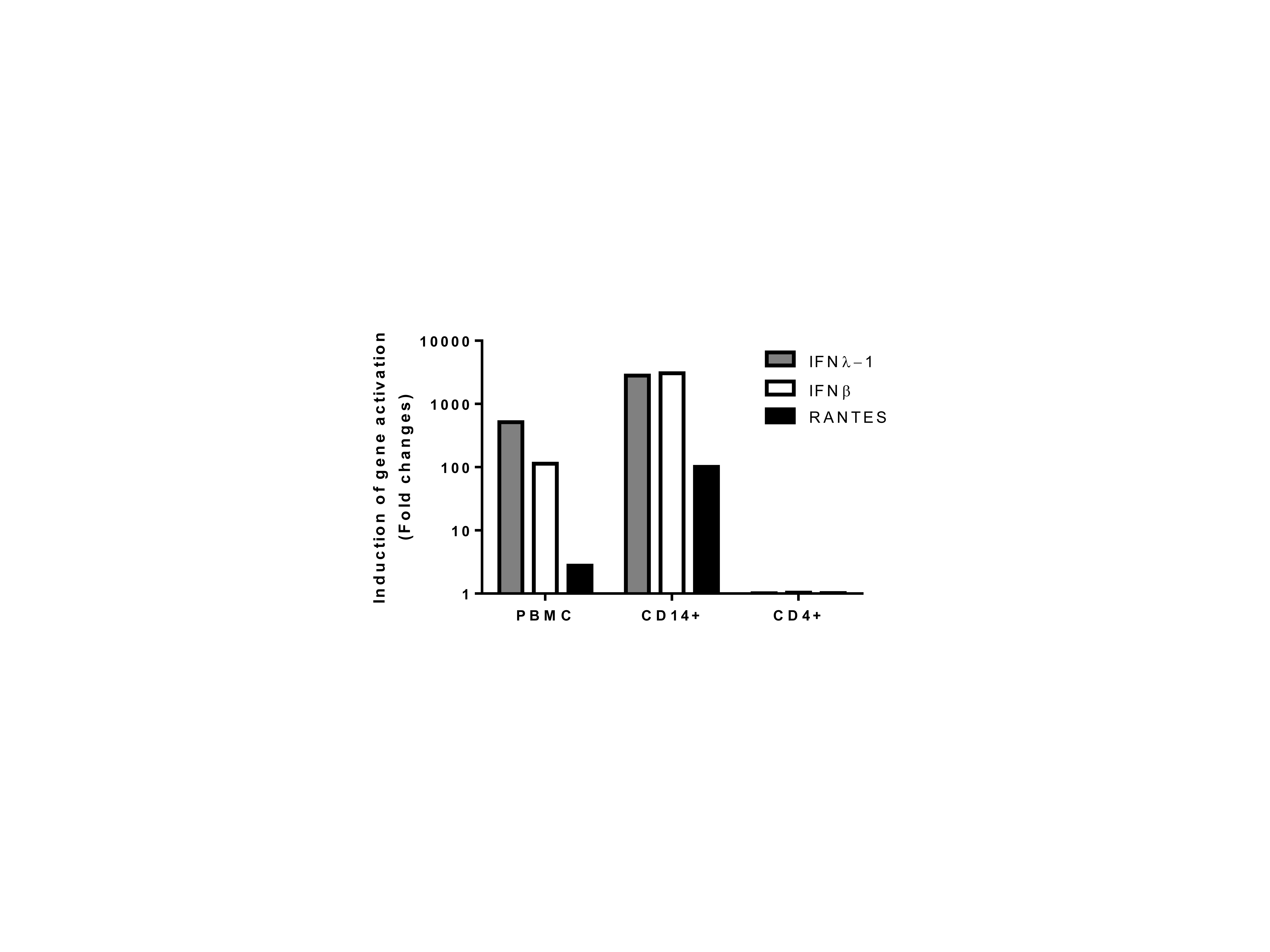

Supplement: Supplementary file [file NIHMS624682-supplement-Supplementary_file.zip › supplemental fig 1.tif]

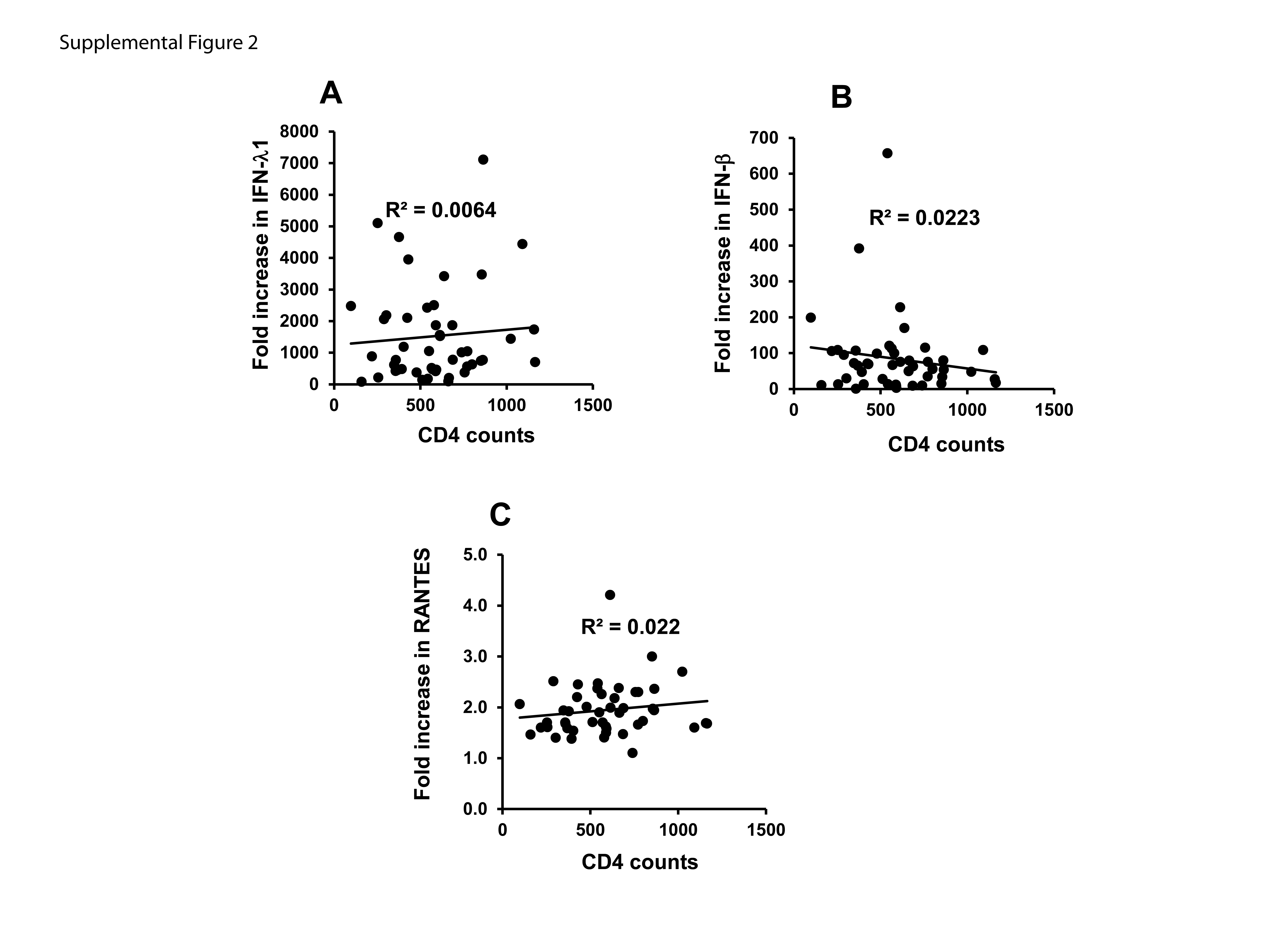

Supplement: Supplementary file [file NIHMS624682-supplement-Supplementary_file.zip › supplementary figure 2.tif]
